# Tubulin expression and modification in heart failure with preserved ejection fraction (HFpEF)

**DOI:** 10.1038/s41598-022-19766-5

**Published:** 2022-09-21

**Authors:** Lisa Schulz, Sarah Werner, Julia Böttner, Volker Adams, Philipp Lurz, Christian Besler, Holger Thiele, Petra Büttner

**Affiliations:** 1 Department of Cardiology, Heart Center Leipzig at University of Leipzig, Strümpellstr. 39, 04289 Leipzig, Germany; 2 Department of Cardiology, University Medicine TU Dresden, Dresden, Germany; 3 Dresden Cardiovascular Research Institute and Core Laboratories GmbH, Dresden, Germany

**Keywords:** Cardiology, Cardiovascular diseases, Heart failure

## Abstract

Diastolic dysfunction in heart failure with preserved ejection fraction (HFpEF) is characterised by increased left ventricular stiffness and impaired active relaxation. Underpinning pathomechanisms are incompletely understood. Cardiac hypertrophy and end stage heart disease are associated with alterations in the cardiac microtubule (MT) network. Increased amounts and modifications of α-tubulin associate with myocardial stiffness. MT alterations in HFpEF have not been analysed yet. Using ZSF1 obese rats (O-ZSF1), a validated HFpEF model, we characterised MT-modifying enzymes, quantity and tyrosination/detyrosination pattern of α-tubulin at 20 and 32 weeks of age. In the left ventricle of O-ZSF1, α-tubulin concentration (20 weeks: 1.5-fold, *p* = 0.019; 32 weeks: 1.7-fold, *p* = 0.042) and detyrosination levels (20 weeks: 1.4-fold, *p* = 0.013; 32 weeks: 1.3-fold, *p* = 0.074) were increased compared to lean ZSF1 rats. Tyrosination/α-tubulin ratio was lower in O-ZSF1 (20 weeks: 0.8-fold, *p* = 0.020; 32 weeks: 0.7-fold, *p* = 0.052). Expression of α-tubulin modifying enzymes was comparable. These results reveal new alterations in the left ventricle in HFpEF that are detectable during early (20 weeks) and late (32 weeks) progression. We suppose that these alterations contribute to diastolic dysfunction in HFpEF and that reestablishment of MT homeostasis might represent a new target for pharmacological interventions.

## Introduction

The prevalence of heart failure with preserved ejection fraction (HFpEF) is increasing and nearly every second patient with heart failure (HF) is diagnosed with HFpEF^1,2^. One of the pathophysiological cornerstones of HFpEF is diastolic dysfunction, characterised by increased myocardial fibrosis, but also increased cellular stiffness^3^. One well-known pathomechanism of cellular stiffness in HFpEF is altered titin phosphorylation, which directly affects myocardial contractility^4^. Titin regulates the passive elasticity in cardiomyocytes and its hypo-phosphorylation may lead to increased passive ventricular stiffness. Nevertheless, the complete titin pathomechanism and other cellular processes responsible for myocardial stiffness are not clarified yet^5^.

Microtubules (MT) are dynamic, hollow structures and the stiffest filaments in cardiomyocyte cytoskeleton. They are formed by polymerisation of the heterodimers α-and β-tubulin powered by hydrolysis of GTP^6^. MT, especially the α-tubulin subunit are subjected to posttranslational modifications like tyrosination and detyrosination that regulate restructuring of the cytoskeleton. Tubulin tyrosin ligase (TTL) adds tyrosine to the C-terminus of free α-tubulin, making it accessible for polymerisation. In contrast, tubulin carboxypeptidase vasohibin 1 (VASH1) removes the tyrosine from α-tubulin, enabling depolymerisation. Thus, tubulin turnover in form of tyrosination and detyrosination is a cyclic event involving α- tubulin subunits^7^.

MT tyrosination and detyrosination play an important role in cardiomyocyte contraction^8^. A well-balanced level of detyrosinated MT is important for proper cardiomyocyte contraction as MT bind to sarcomeres and form a physiological resistance for contraction^9^. In patients with hypertrophic and dilated cardiomyopathies and end stage heart failure higher amount of MT and detyrosinated α-tubulin were described. Further, denser MT network and high levels of detyrosinated α-tubulin are associated with impaired cardiomyocyte contraction and increased resistance^10^.

An imbalance in MT restructuring and modification in HFpEF was not analysed yet. Although, it may represent an underlying pathomechanism of increased myocardial stiffness and impaired diastolic function. The availability of human myocardial tissue, especially from HFpEF patients, is very limited. In addition, the progression of HFpEF in humans cannot be determined on a molecular level. Therefore, we used ZSF1 obese rats, an accepted animal model for HFpEF^11–13^. Obese ZSF1 (O-ZSF1) hybrid rats are compound heterozygote with two different mutated alleles coding defective leptin receptor, resulting in loss of the receptor function. Thus, these animals have increased food intake and develop pronounced features of obesity, diabetes, and resultant HFpEF. ZSF1 rats that have inherited two wild type alleles or only one of the parental mutated alleles, have a normal leptin signalling, balanced food intake and energy expenditure, normal weigth, and do not develop disease traits (L-ZSF1). In the present study, we measured protein- and gene expression of MT associated proteins in O-ZSF1 with HFpEF and L-ZSF1 rats without HFpEF to address the mechanism of MT modifications in HFpEF for the first time.

## Results

### O-ZSF1 rats characteristics for HFpEF

O-ZSF1 rats had significantly higher body weights (*p* < 0.001 for both 20 and 32 weeks) as well as increased heart weight (20 weeks: *p* < 0.001; 32 weeks: *p* = 0.002) compared to L-ZSF1 rats. The left ventricular (LV) ejection fraction (LVEF) in O-ZSF1 rats was > 50% and comparable with L-ZSF1. Also, heart rate was comparable in L-ZSF1 and O-ZSF1 at both time points. Echocardiographic and laboratory parameters compatible with the HFpEF phenotype in O-ZSF1 were: increased E/e′ (20 weeks: 1.4-fold, *p* < 0.001; 32 weeks: 1.25-fold, *p* = 0.158), increased LV enddiastolic volume (LVEDV) (20 weeks: 1.3-fold-change, *p* < 0.001; 32 weeks: 1.6-fold change, *p* < 0.002), altered NT-proBNP (20 weeks: 1.3-fold increased, *p* = 0.047; 32 weeks: 1.3-fold decreased, *p* = 0.051), and increased LV diastolic diameter (LVDd) (20 weeks: 1.2-fold, *p* < 0.001; 32 weeks: 1.13-fold, p = 0.006) (Table 1).

**Table 1:** Characteristics of O-ZSF1 and L-ZSF1 rats at 20 and 32 weeks of age.

	20 weeks			32 weeks		
	O-ZSF1	L-ZSF1	*p*-value	O-ZSF1	L-ZSF1	*p*-value
Body weight [g]	468 ± 28	235 ± 9	< 0.001	529.2 ± 23.1	283.4 ± 8.8	< 0.001
Heart weight [g]	1.38 ± 0.07	0.93 ± 0.05	< 0.001	1.56 ± 0.15	1.16 ± 0.12	0.002
Heart rate [bpm]	214 ± 17	216 ± 18	0.812	222 ± 9	220 ± 22	0.839
E/e′	21.7 ± 3.6	15.0 ± 2.8	< 0.001	22.4 ± 2.5	17.8 ± 4.9	0.158
LVEF [%]	63 ± 15	55 ± 12	0.140	53.1 ± 5.9	58 ± 6.3	0.219
LVEDV [cm^3^]	0.60 ± 0.11	0.46 ± 0.04	< 0.001	0.97 ± 0.16	0.6 ± 0.062	0.002
LVDd [cm]	0.94 ± 0.11	0.79 ± 0.06	< 0.001	0.75 ± 0.05	0.66 ± 0.04	0.006
NT-proBNP [pg/ml]*	1200 ± 338	895 ± 371	0.047	266 ± 64	340 ± 49	0.051

Mean and standard deviation are shown; *p*-value was calculated using Student’s t-test. Bpm (beats per minute), E/e′ (ratio of mitral peak velocity of early filling (E) to early diastolic mitral annular velocity (e′)), LVEF (left ventricular ejection fraction), LVEDV (left ventricular end-diastolic volume), LVDd (left ventricular diastolic diameter), NT-proBNP (N terminal B natriuretic peptide).

*Due to different manufacturers, the results at 20 and 32 weeks are not directly comparable.

### Quantification of α-tubulin, its post-translational modification

**Figure 1:**
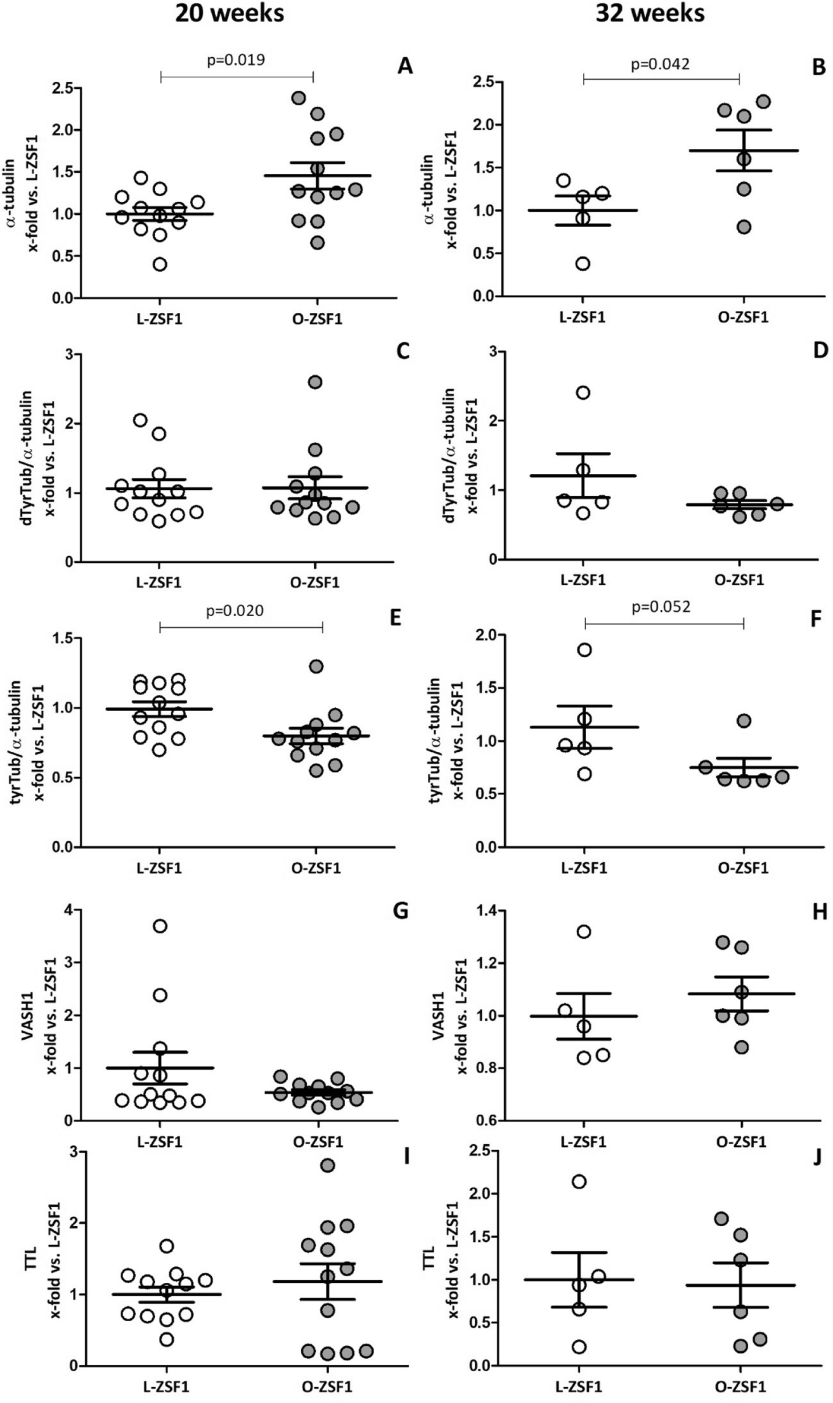
Protein expression of α-tubulin (**A**/**B**), ratios of detyrosinated tubulin/α-tubulin (dtyrT/α-tubulin) (**C**/**D**), tyrosinated tubulin/α-tubulin (tyrTub/α-tubulin) (**E**/**F**) and enzymes involved in tubulin detyrosination cycle namely tubulin carboxypeptidase vasohibin 1 (VASH1) (**G**/**H**) and tubulin tyrosin ligase (TTL) (**I**/**J**) measured in left ventricle tissue of O-ZSF1 and L-ZSF1 rats at an age of 20 weeks (**A**/**C**/**E**/**G**/**I**) and 32 weeks (**B**/**D**/**F**/**H**/**J**). Y-axis shows arbitrary units of protein expression normalised to GAPDH (for α-tubulin, detyrosinated and tyrosinated α-tubulin) or Histone H2B (for VASH1 and TTL). Further, measurements were normalised to the mean signal of all L-ZSF1 rats. Scatter plots represent the median, 25th and 75th percentiles. *p*-values were calculated using Student’s t-test (for normal distribution) and non-parametric Mann–Whitney-*U*-test.

Western Blots were performed for quantitative protein detection of α-tubulin, its post-translational modification forms tyrosinated and detyrosinated tubulin and associated enzymes in LV tissue [see supplement for original Western Blots (Supplementary Figs. 3A–E, 4A–E) and example of ROI determination (Supplementary Fig. 2)]. We observed significantly increased amounts of α-tubulin in LV of O-ZSF1 rats compared to L-ZSF1 (20 weeks: 1.5-fold, *p* = 0.019; 32 weeks: 1.7-fold, *p* = 0.042) (Fig. 1A,B). Furthermore, we detected higher amounts of detyrosinated tubulin in O-ZSF1 compared to L-ZSF1 (20 weeks: 1.4-fold, *p* = 0.013; 32 weeks: 1.3-fold, *p* = 0.074). The ratio of detyrosinated tubulin to total α-tubulin (dTyrTub/α-tubulin) was comparable between O-ZSF1 and L-ZSF1, (20 weeks: onefold, *p* = 0.921; 32 weeks: 0.7-fold, *p* = 0.227) (Fig. 1C,D). Tyrosinated tubulin was comparable but the ratio of tyrosinated tubulin to total α-tubulin (tyrTub/α-tubulin) was lower in O-ZSF1 rats (20 weeks: 0.8-fold, *p* = 0.020; 32 weeks: 0.7-fold, *p* = 0.052) (Fig. 1E,F). No significant difference for Vasohibin 1 (VASH1), the enzyme removing tyrosine from α-tubulin (20 weeks: 0.5-fold, *p* = 0.681; 32 weeks: 1.1-fold, *p* = 0.457) (Fig. 1G,H) as well as for tubulin tyrosine ligase (TTL) (20 weeks: 1.2-fold, *p* = 0.512; 32 weeks: 0.9-fold, *p* = 0.883) (Fig. (1I,J) was detected comparing O-ZSF1 and L-ZSF1.

All measurements were also done in septum and RV and no differences between the experimental groups were observed for any of the analytes or ratios reported above.

### Expression of genes related to tubulin modifications

**Figure 2:**
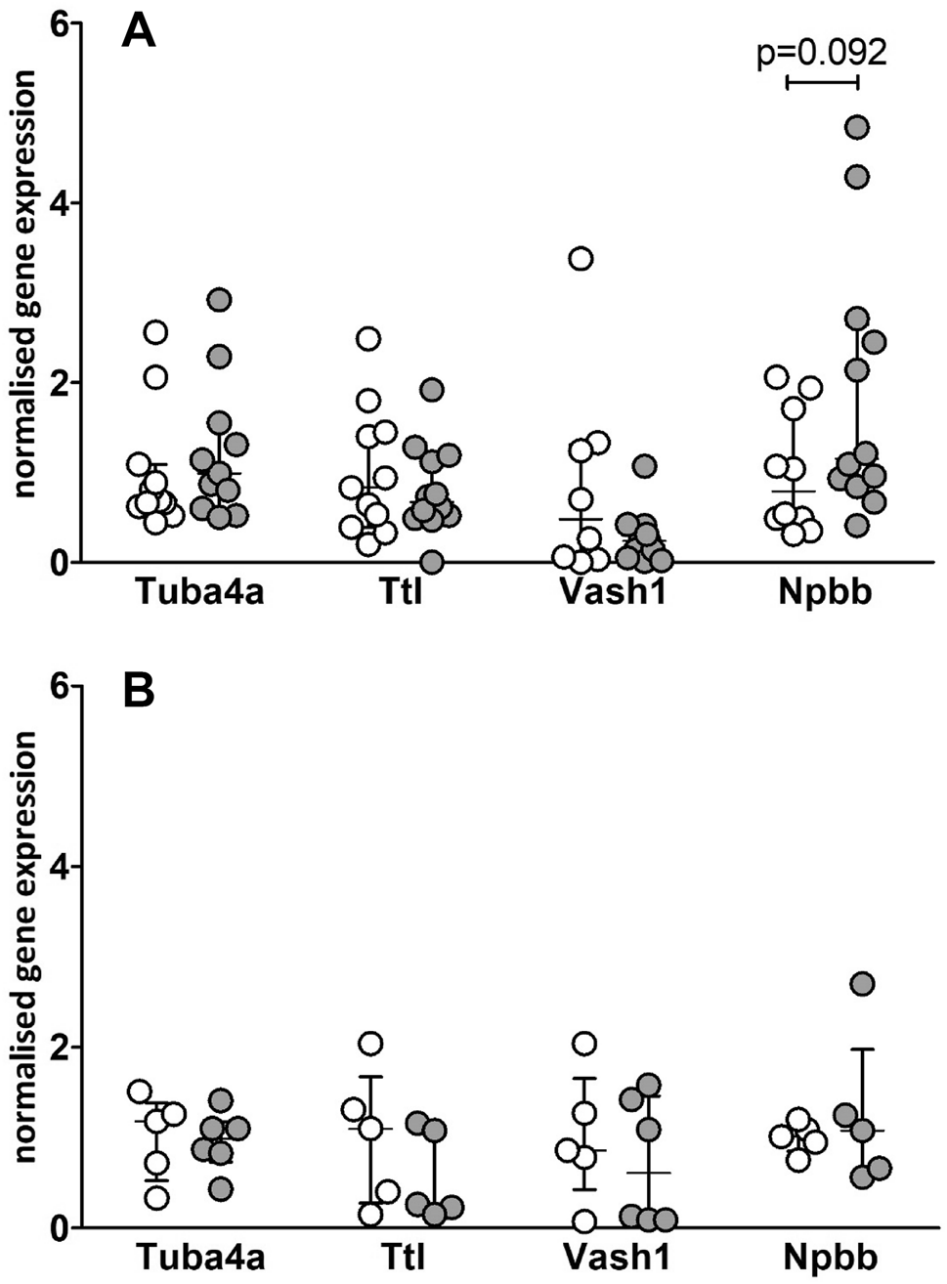
Gene expression of α-tubulin a4a (Tuba4a), tubulin tyrosine ligase (Ttl), vasohibin1 (Vash1) and NT-proBNP (Npbb) in left ventricle tissue of L-ZSF1 (white circles) and O-ZSF1 (grey circles) after 20 weeks (**A**) and 32 weeks (**B**). Y-axis shows arbitrary units of gene expression normalised to Hprt1 and to the mean signal of all L-ZSF1 rats. Scatter plots represent the median, 25th and 75th percentiles. *p*-values were calculated using Student’s t-test (for normal distribution) and non-parametric Mann–Whitney-U-test.

Gene expression of Tuba4a (coding for α-tubulin a4a), Ttl (coding for tubulin tyrosine ligase), Vash1 (coding for vasohibin1) and Npbb (coding for NT-proBNP) in LV tissue was detected by quantitative RT-PCR (Fig. 2). Tuba4a was comparable in O-ZSF1 and L-ZSF1 rats (20 weeks: 1.2-fold, *p* = 0.411; 32 weeks: 1.04-fold, *p* = 0.783). Ttl was comparable in O-ZSF1 and L-ZSF1 rats (20 weeks: 1.2-fold, *p* = 0.454; 32 weeks: 1.7-fold, *p* = 0.345). For Vash1 no differences were detected (20 weeks: 3.1-fold, *p* = 0.432; 32 weeks: 1.3-fold, *p* = 0.927). Npbb was 1.8-fold higher in O-ZSF1 rats at an age of 20 weeks (*p* = 0.092) and at an age of 32 weeks it was comparable (0.8-fold, *p* = 1.000) with L-ZSF1 rats.

## Discussion

In this study protein and gene expression of tubulin, its modified forms and enzymes involved in tubulin turnover were analysed in LV tissue of O-ZSF1 rats, an animal model for HFpEF.

Increased protein concentrations of α-tubulin in O-ZSF1 rats at an age of 20 weeks and 32 weeks were found. While this increased protein concentration was not described for HFpEF before, it is well known that α-tubulin content in LV tissue is higher in chronic heart failure and cardiac hypertrophy^14,15^. Higher MT stability due to higher tubulin synthesis or turnover underpinning myocardial dysfunction was also described in LV pressure overload hypertrophy^16^.

Detyrosination of α-tubulin plays an important role in cardiac function^17^. In cardiomyocytes, detyrosinated MT are coupled to the sarcomeres and form a physiological resistance by buckling during contraction. A reduction of MT detyrosination thus disturbs this interaction, resulting in less MT buckling, whereby sarcomeres shorten and stretch with less resistance^10^. However, an increase of detyrosinated MT results in a higher level of resistance, causing myocardial stiffness^8,10,18^. Our results show that increased amounts of detyrosinated MT are detectable in O-ZSF1 compared to L-ZSF1 rats. As α-tubulin was also increased, the ratio of dtyrTub/α-tubulin was comparable between both experimental groups. Nevertheless, as the general content of dtyrTub is higher in O-ZSF1 LV, increased resistance and stiffness are plausible. Moreover, we detected a decreased ratio of tyrTub/α-tubulin in O-ZSF1 rats, what further underpins an imbalance of total tyrosinated and detyrosinated α-tubulin.

Up to now, increased passive stiffness in HFpEF was mainly attributed to concentric remodeling, titin isoform switch and titin hypophosphorylisation^4^. Based on our findings we postulate that an increase in detyrosinated MT, as observed in O-ZSF1 rats, may also contribute to left ventricular stiffness and impaired diastolic performance in HFpEF.

We tried to identify regulatory mechanisms that account for the observed alterations. Firstly, gene expression of Tuba4a (coding for α-tubulin a4a), as it is the only α-tubulin which is already translated in its detyrosinated form was analysed^19^. No differences were observed and we assume that altered α-tubulin a4a amounts do not explain the observations.

C-terminal detyrosination of α-tubulin is enzymatically driven by tubulin carboxypeptidases vasohibin 1 and 2, whereas VASH1 is specific for cardiomyocytes^20^. VASH1 protein content in LV tissue of O-ZSF1 rats was comparable to L-ZSF1 rats. TTL, which adds the tyrosine to α-tubulin, was also found unchanged. Also, the gene expressions of both enzymes were unchanged. Therefore, we conclude that the function of VASH1 and TTL is not regulated on translational or transcriptional level. Interestingly, it has been shown that VASH1 is stabilised by the chaperone small vasohibin-binding protein and that this interaction has a regulatory impact on MT associated spindle function and mitosis^21^. This should be analysed in further studies.

All analyses were also done in septum and RV without detecting any differences. It is controversially discussed whether HFpEF is primarily affecting LV and if RV is initially mechanically affected without LV-specific pathomechanisms being present in the first place. Our observations indicate that at least disturbed MT homeostasis in O-ZSF1 is LV specific but a possible effect on LV mechanics could affect RV indirectly.

Serum concentrations of NT-proBNP were significantly higher in O-ZSF1 than in L-ZSF1 rats at an age of 20 weeks, while LV gene expression of NT-proBNP (encoded by Npbb) was increased by trend. At an age of 32 weeks, gene expression was comparable between the experimental groups and serum concentrations were by trend lower in O-ZSF1 than in L-ZSF1 rats. These results are in contrast to recently published results^12^. Although, they are in line with the hypothesis that the wall tension of the concentrically remodeled LV is lower in late HFpEF compared to early progression stages due to only episodically increased filling pressures^3^. Accordingly, in humans natriuretic peptides are higher in acute HF than in chronic HF where they range within normal values and are of limited predictive use^22^. Importantly, BNP levels are sensitive to obesity. BNP is removed from circulation by binding to natriuretic peptide clearance receptor that is particularly strongly expressed in adipocytes^5^. Buckley et al. showed in a cohort of obese HFpEF patients that 50% had NT-proBNP levels below the diagnostic threshold^23^.

Disturbed MT homeostasis may represent a pharmaceutical target. It was shown that suppression of detyrosinated MT via overexpression of TTL in rat and human cardiomyocytes has a positive impact on myocardial contractility and improves dyastolic function^12,26^. It has to be explored whether these and other treatments have a beneficial effect on detyrosinated α-tubulin and contractility in HFpEF. An increase of detyrosinated α-tubulin using parthenolide in hypertrophic cardiomyopathy was described and is still disputed^24,25^. Colchicine, an anti-inflammatory drug and mitosis inhibitor, which directly acts on MT was discussed for the treatment of heart failure with reduced ejection fraction (HFrEF) but did not improve left ventricular function^26^. Nevertheless, it may be useful in HFpEF treatment.

We postulate that higher protein concentrations of α-tubulin and its modifications are involved in altered LV stiffness and impaired diastolic performance in HFpEF. α-tubulin and its detyrosinated form may represent a potential treatment target in HFpEF, which is urgently needed. Therefore, we are planning inhibition experiments to investigate whether α-tubulin and its detyrosinated form have an impact on myocardial stiffness. Especially the mechanism of alterations in total α-tubulin and its detyrosinated form in contrast to comparable expression of α-tubulin modifying enzymes is of interest. These experiments may contribute to understanding of the mechanism of e.j. the chaperone small vasohibin-binding protein which stabilises VASH1.

For further research, it is also of interest how the detyrosination and tyrosination of α-tubulin affects the interaction of MT binding proteins. The MT binding protein dynein and its cofactor dynactin act as MT motorprotein transporting cellular cargos^27^. McKenney et al.^28^ showed that the motility of the dynein-dynactin complex increases with the rate α-tubulin tyrosination. We hypothesize that the motility of the dynein-dynactin complex may be reduced in HFpEF.

Moreover, HFpEF is associated with inflammatory endothelial activation and oxidative stress. Lower myocardial nitrite/nitrate concentrations^29^ and reduced concentrations of nitrite in O-ZSF1 rats as well as significant lower concentrations of Arginin and Homoarginin in heart and serum are described in previous experiments^30^. Its known that oxidative stress impairs MT polymerisation^31^. Therefore it is interesting whether there is also a potential connection between oxidative stress and the tyrosination/detyrosination of α-tubulin.

## Limitations

Our study included only few animals aged 32 weeks. We thus assume that because of the heterogeneity in this group small effects might have been missed. We observed numerically alterations in regulatory processes that may reach significance in larger experimental groups. For instance, the E/e′ within O-ZSF1 rats decreased after 32 weeks, which could also be due to the small number of animals. In addition, complications occur in O-ZSF1 rats at 32 weeks of age that exacerbate the phenotype of HFpEF through loss of skeletal muscle function and aortic valve sclerosis. Whether and to what extent this influences tubulin regulation cannot be answered within the scope of this study.

Analysis of potential morphological changes in tubulin distribution was not done in this study due to limitations in sample material. Further experiments should include immunohistological or electron microscocopy to address this point.

The transferability of data from animal models to humans is limited when it comes to the effects of older age; comorbidities, medication commonly used in human patients, hormone status or lifestyle (e.g. smoking or exercise). Findings from animal experiments should thus always be validated using human material. For example, the PARAGON-HF study clearly showed that the values for LVDd and LVEDVi can be within the normal range despite the presence of HFpEF^32^. Nevertheless, animal models like the ZSF1 HFpEF rat model are hypothesis generating and may help to elucidate yet unknown pathomechanisms.

An association of altered tubulin levels and modifications with characteristics of cardiomyocyte mechanobiology is necessary but was beyond the scope of this study.

## Methods

### ZSF1 rats as HFpEF animal model

Animal care and procedures were performed in accordance with ARRIVE guidelines and relevant animal welfare guidelines and regulations and were approved by the local Animal Research Council, University of Leipzig and the Landesbehörde Sachsen (TVV 30/18, TVV 40/19).

Female ZSF1 hybrid rats crossed between a Zucker diabetes fatty female, and a spontaneous hypertensive heart failure male rat were used as animal model (ZSF1-Lepr^fa^ Lepr^cp^/Crl, Charles River, Indianapolis, USA). We analysed twelve O-ZSF1 (obese) and L-ZSF1 (lean) rats respectively aged 20 weeks, and six O-ZSF1 rats and five L-ZSF1 rats at 32 weeks of age. All animals were kept at identical conditions under a 12:12 h light/dark cycle with food and water provided ad libitum. Standard chow rich in energy and protein content was delivered by Ssniff (Soest, Germany). Body weight and food intake were recorded every week. Non-invasive echocardiography (Vivid-J, GE Healthcare, Chicago, USA and Prospect Sharp T1, Scintica Instrumentation Inc., Maastricht, Netherlands) was used to confirm the presence of HFpEF. Animals were sacrificed by exsanguination.

### Sample preparation

Myocardial tissue was weighted before separation into LV, RV and septum. Samples were immediately snap-frozen in liquid N_2_ and stored at − 80 °C until further use. Frozen samples were pulverised and used for RNA and protein analysis. RNA isolation using up to 30 mg of tissue was done with the RNeasy Kit and QIAShredder (Qiagen, Hilden, Germany) according to the manufacturer’s recommendation. RNA quality was determined on Fraqment Analyzer (Agilent, Santa Clara, USA) and evaluated with PROsize 2.0 Software. RNA quality numbers ranged between eight and ten (ten indicates highest quality RNA). RNA concentration was photometrically determined, and 200 ng RNA was reverse transcribed using Omniscript RT kit with oligo dT Primer (Qiagen, Hilden, Germany). For protein analysis 10–20 mg of frozen sample were lysed in RIPA buffer (10 mM Tris–HCl, pH 8.0, 1 mM EDTA, 0.5 mM EGTA, 1% Triton X-100, 0.1% sodium deoxycholate, 0.1% SDS, 140 mM NaCl) containing a protease and a phosphatase inhibitor mix (Serva, Heidelberg, Germany) and sonicated. Protein concentration was analysed using the BCA method (bicinchoninic acid assay, Pierce, Bonn, Germany). Blood was withdrawn at sacrifice by direct puncture of the heart before it stopped beating.

### Western blot analysis

For determination of protein expression 12% SDS–polyacrylamide gels were loaded with 20 μg of protein before transferring to a polyvinylidene fluoride membrane. Membranes were blocked with 5% milk (blotting grade, Roth, Germany), incubated overnight at 4 °C with primary antibodies and rinsed with 1 × TTBS 3 × 10 min. Peroxidase-conjugated secondary antibodies in 1% milk were incubated for one hour and specific bands were visualised by enzymatic chemiluminescence (Super Signal West Pico, Thermo Fisher Scientific Inc., Bonn, Germany).

Densitometry of each specific band was quantified using a semi-automated 1D scan software package (Vision-Capt, Vilber Lourmat, Eberhardzell, Germany). Each band was manually marked and densitometry was automatically determined [see supplement for original Western Blots (Supplementarys Fig. 3A–E, 4A–E) and example of ROI determination (Supplementary Fig. 2)]. Amounts of α-tubulin (Abcam ab7291, 1:10 000), tyrosinated tubulin (Sigma T9028, 1:400), detyrosinated tubulin (Millipore ab3201, 1:500), TTL (Proteintech 13618-1-AP, 1:1000) and VASH1 (Abcam ab199732, 1:1000) were determined. For normalisation of α-tubulin (50 kDa), tyrosinated (55 kDa) and detyrosinated (55 kDa) tubulin we used glyceraldehyde-3-phosphate dehydrogenase (GAPDH; 37 kDa) (HyTest 5G4, 1:30 000) and for normalisation of TTL (43 kDa) and VASH1 (41 kDa) we used acetylated histone 2B (H2B, 17 kDa) (Elabscience E-AB-66503, 1:500). We used peroxidase conjugated secondary antibodies (Sigma POD 9044 anti-mouse, 1:5000 or Millipore AP187P anti-rabbit, 1:10,000; depending on the host species of the primary antibody). All protein amounts were further normalised to the mean level in L-ZSF1 control group.

### Quantitative realtime-PCR

Quantitative Realtime-PCR was performed to detect gene expression of Tuba4a, Vash1, Ttl, Npbb in O-ZSF1 and L-ZSF1 rats. We used Takyon NoRox Sybr Mastermix Blue (Eurogentec, Lüttich, Belgium) on a BioRad CFX system (BioRad, Hercules, USA). Primers were designed exon-spanning with an annealing temperature of 60 °C (sequences in Table 2). For estimation of copy numbers and determination of reaction efficiency, we used standard curves. All samples were measured in triplicates and specimens with standard deviation > 0.250 were excluded. Hypoxanthine phosphoribosyltransferase 1 (Hprt1) was tested, determined to be a stable housekeeping gene, and used for expression normalization of the target genes. Furthermore, the expression of target genes in O-ZSF1 was normalized to L-ZSF1 rats.

**Table 2:** Primers used for quantitative realtime PCR.

Gene	Forward primer	Reverse primer
Tuba4a	CAACTATGCCCGTGGTCACT	CTACAGCCGTGGACACTTGT
Vash1	CGCCCTCTACCCTTGAATCC	TGACCCACAGCGATCTAGGA
Ttl	ATCCCTTGAGCGGTTTCTGG	GGACAGCATCACGGAAGGAA
Npbb	CGGATCCAGGAGAGACTTCG	AAAACAACCTCAGCCCGTCA
Hprt1	CCCAGCGTCGTGATTAGTGA	GGCCTCCCATCTCCTTCATG

Gene sequences of Tuba4a (coding for α-tubulin a4a), Ttl (coding for tubulin tyrosine ligase), Vash1 (coding for vasohibin1) and Npbb (coding for NT-proBNP) in LV tissue was detected by quantitative RT-PCR.

### Investigation on NT-proBNP

N-terminal pro Brain Natriuretic Peptide (NT-proBNP) was measured in serum using ELISA assays according to the manufacturer’s recommendations (abx576280, Hölzel Diagnostika, Cologne, Germany and abx256287, Hölzel Diagnostika, Cologne, Germany). Due to different manufacturers, the two assays are not directly comparable. Due to sample limitations, it was not possible to repeat the measurements for all animals using the same assay.

### Statistical analysis

Data are presented as means ± standard deviation. P-values were calculated using Student’s t-test (for normal distribution) and non-parametric Mann–Whitney-U-test. Figures were done using GraphPad Prism 8 (GraphPad Software, San Diego, USA). *p*-value < 0.05 was considered statistically significant and *p*-value < 0.1 was regarded as a trend.

## Supplementary Information

Supplementary Figures.

## Data Availability

All data in the paper are included in the Supplementary Materials. Additional data related to this paper can be available upon the communication with the corresponding author.
