# Supplementary material for: Tubulin expression and modification in heart failure with preserved ejection fraction (HFpEF)
Source: Sci Rep. 2022 Sep 21;12:15734. doi: 10.1038/s41598-022-19766-5 (PMC9492725; doi:10.1038/s41598-022-19766-5)
Supplement: Supplementary file 1 — Supplementary Figures. [file 41598_2022_19766_MOESM1_ESM.docx]

**Supplementary data**

**Figure 1:** For determination of protein size, we used Thermo Scientific PageRuler Plus Prestained Protein Ladder, 10 bis 250 kDa (Fisher Scientific, Schwerte, Germany).


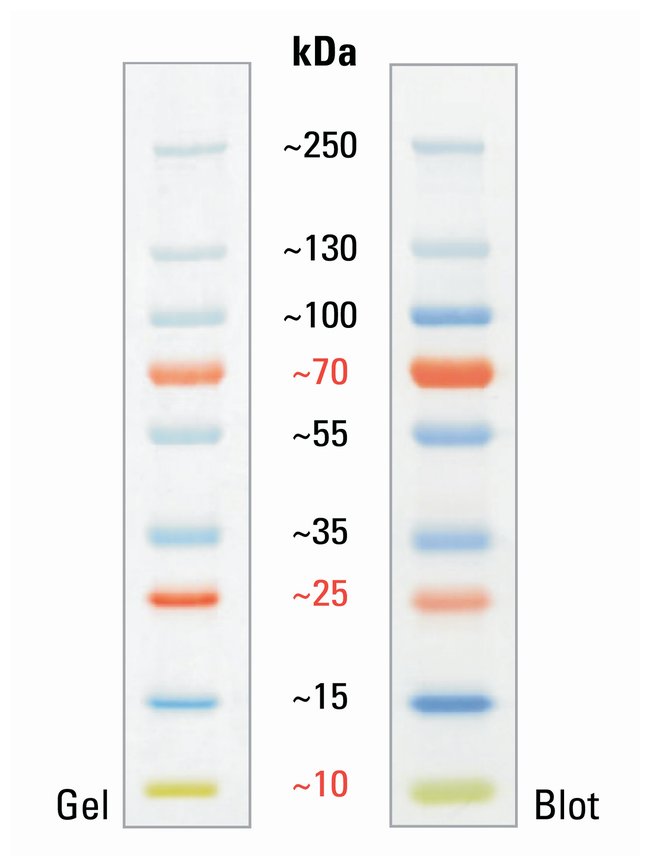


**Figure 2**: Original Western Blot image of α-tubulin in left ventricles of 20 week old O-ZSF1 (n=12) and L-ZSF1 (n=12). Upper panel shows an overlay picture of a bright field recording to visualize the protein ladder and the protein bands recorded in chemiluminescence channel. In the lower panel only the chemiluminescence signal is shown as it provides better contrast for determination of signal intensity. A region of interest (ROI) was manually assigned to enable for calculation of signal intensity (green boxes).

L-ZSF1

O-ZSF1


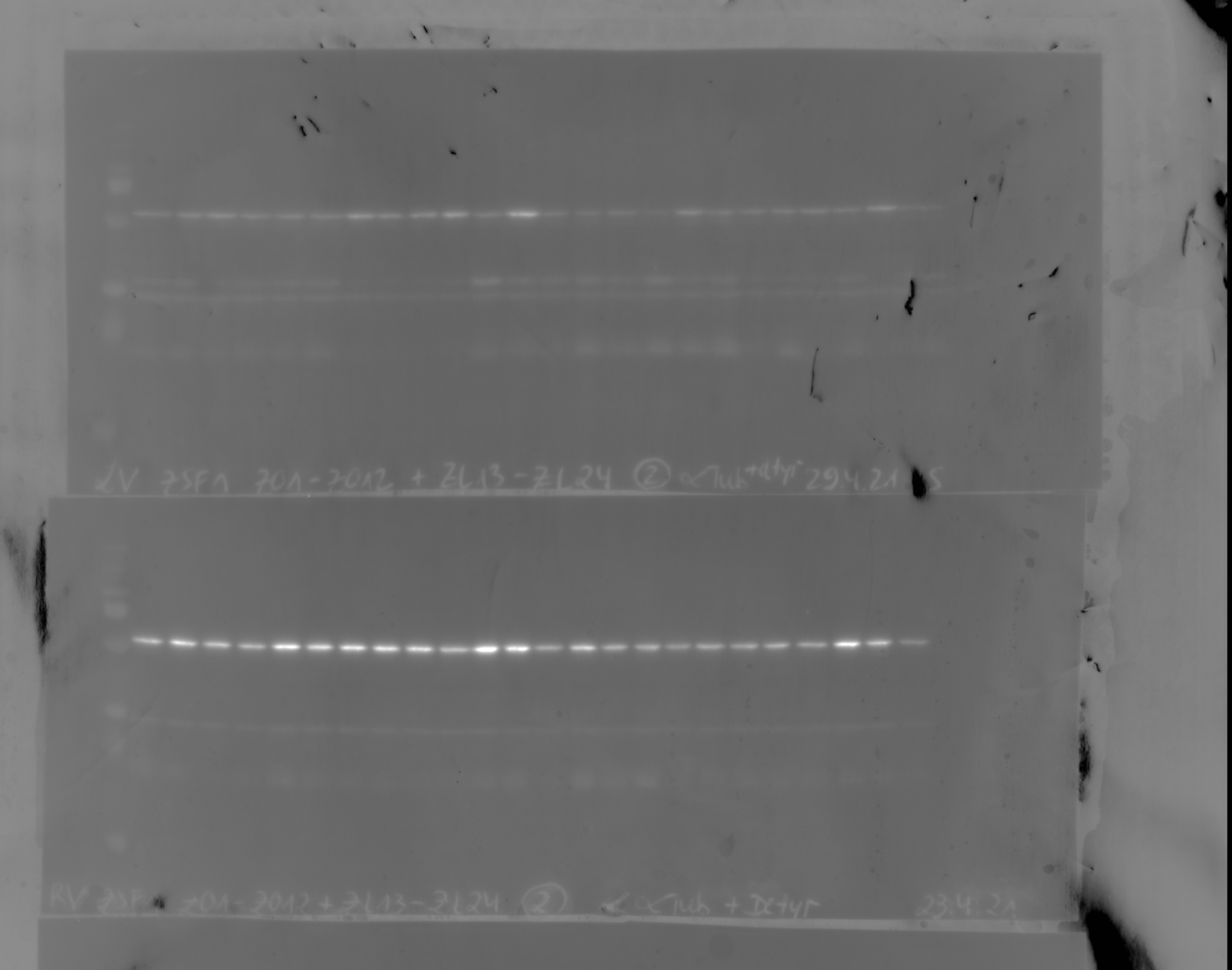


🡨 α-Tub (50 kDa)


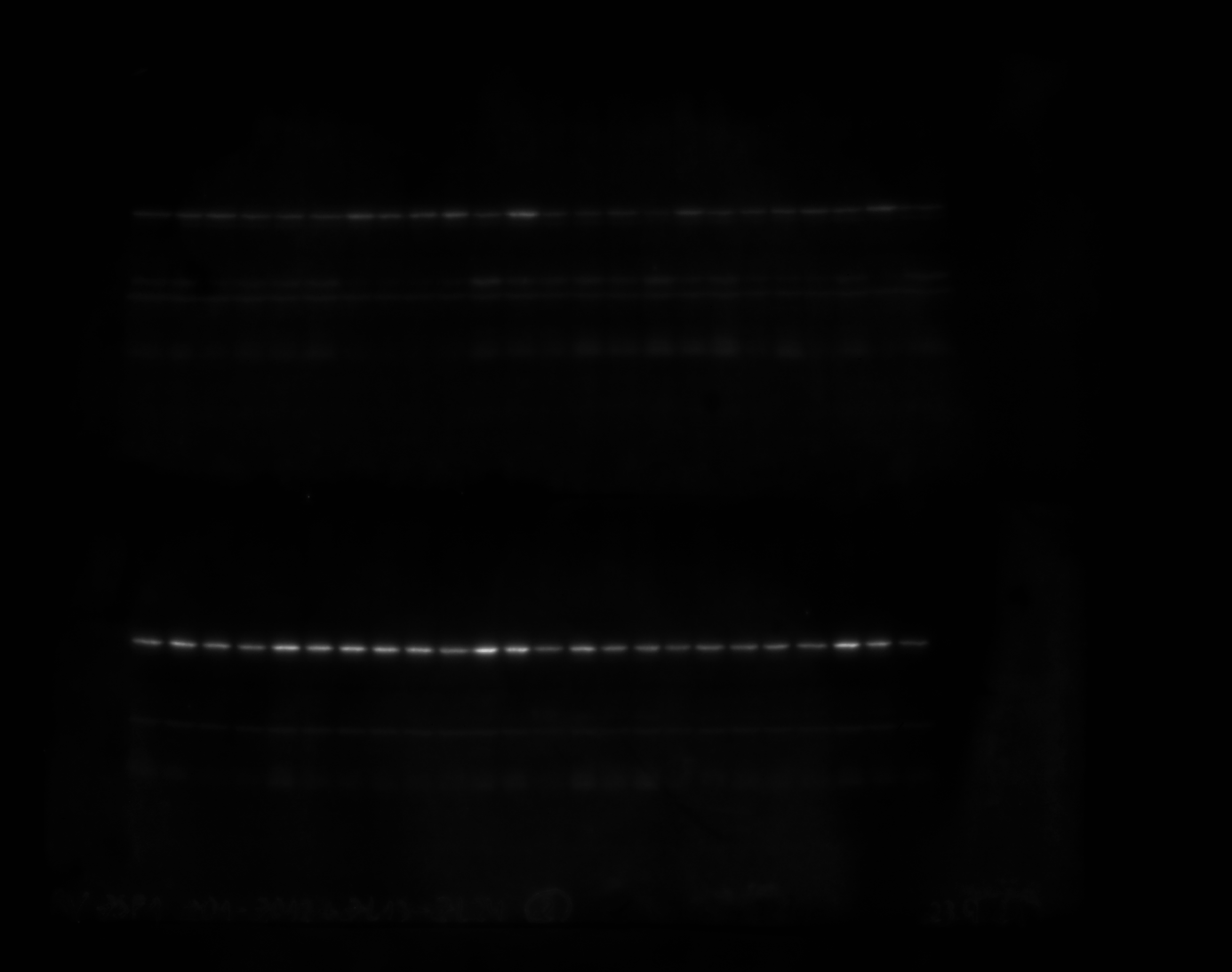


🡨 α-Tub (50 kDa)

L-ZSF1

O-ZSF1

α-tubulin

O-ZSF1

L-ZSF1


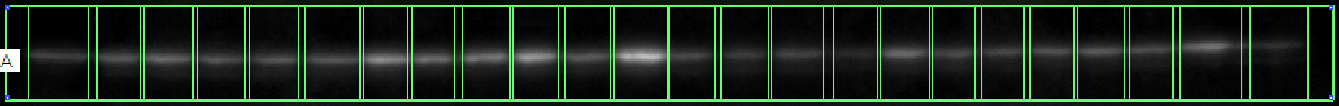


**B**

**Figure 3**: Original Western Blot images of analyses of left ventricles of 20 week old O-ZSF1 (n=12) and L-ZSF1 (n=12) rats (A-E). A - upper panel: α-tubulin (α-Tub), A - lower panel: GAPDH was determined for normalization of α-tubulin, B - upper panel: tyrosinated α-tubulin (tyr α-Tub), B – lower panel: GAPDH, C – upper panel: detyrosinated α-tubulin (detyr α-Tub), C – lower panel: GAPDH, D: VASH1 and H2B (GAPDH determination was not possible because of comparable protein size with VASH1), E: TTL and H2B.

**A**

L-ZSF1

O-ZSF1

**
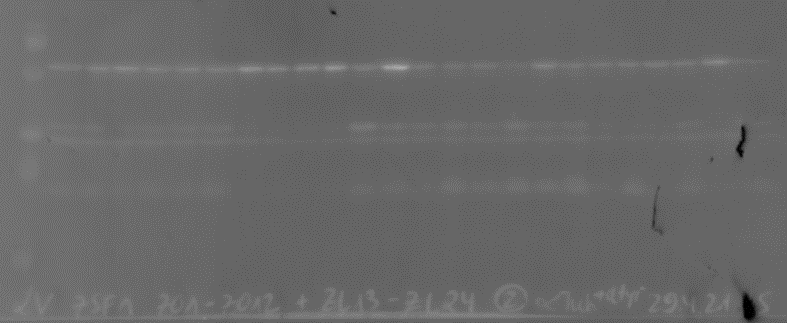
**

🡨 α-Tub (50 kDa)


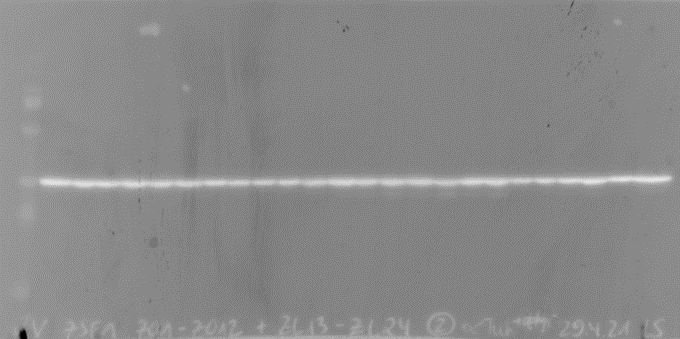


L-ZSF1

O-ZSF1

🡨 GAPDH

(37 kDa)

- 🡨 GAPDH (37 kDa)


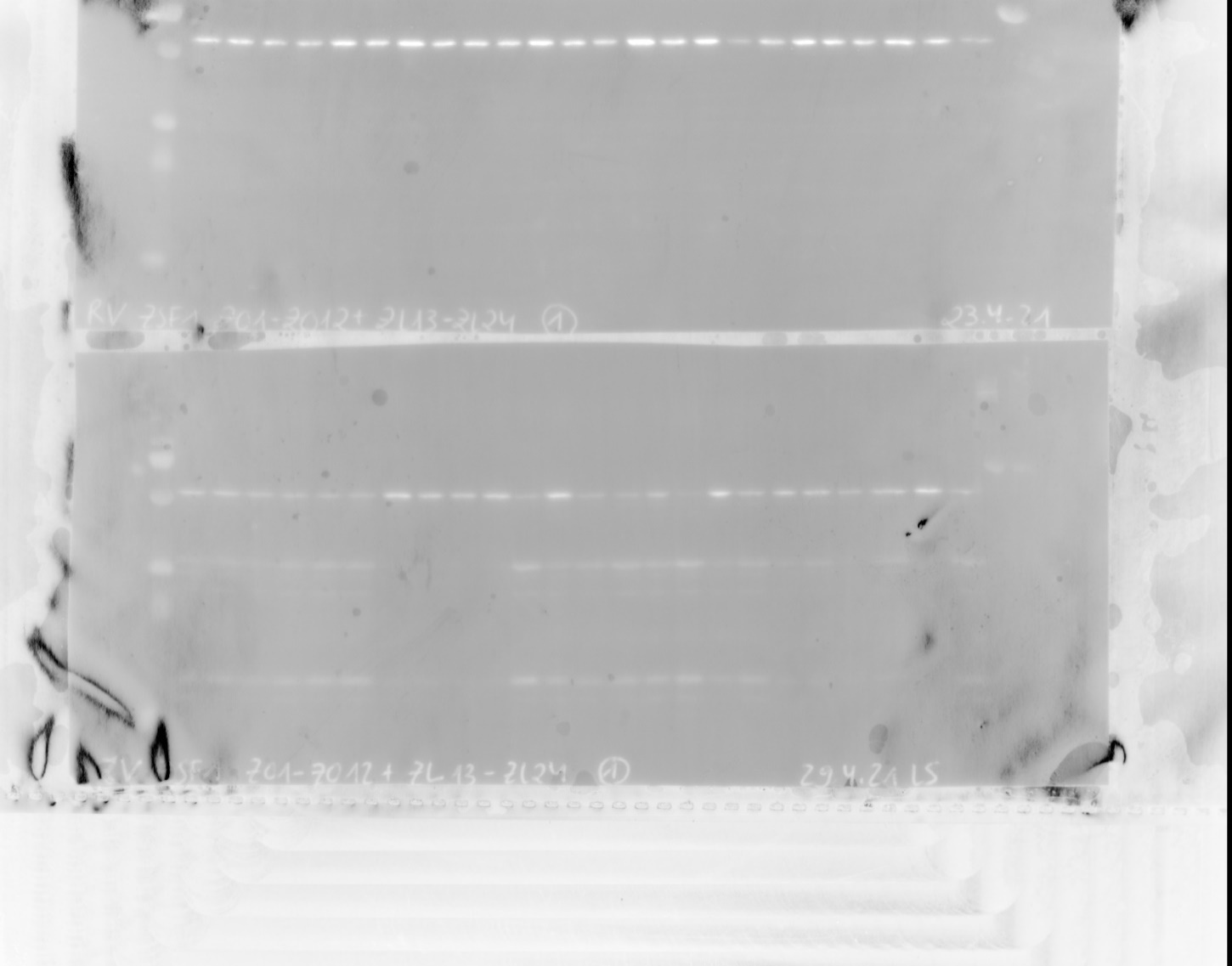
**B**

L-ZSF1

O-ZSF1


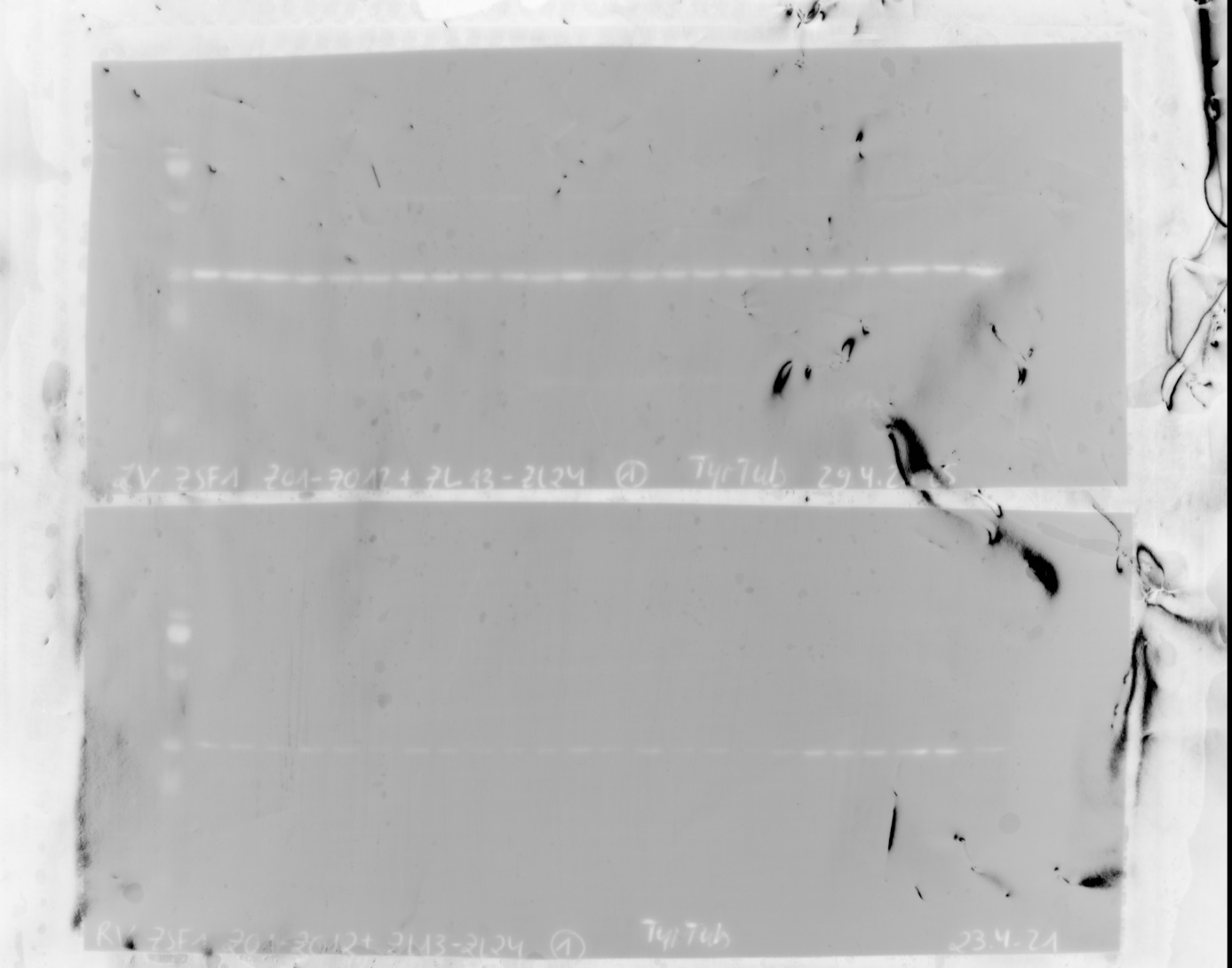
🡨 tyr α-Tub (55 kDa)

L-ZSF1

O-ZSF1

🡨 GAPDH

(37 kDa)

**C**

O-ZSF1

L-ZSF1


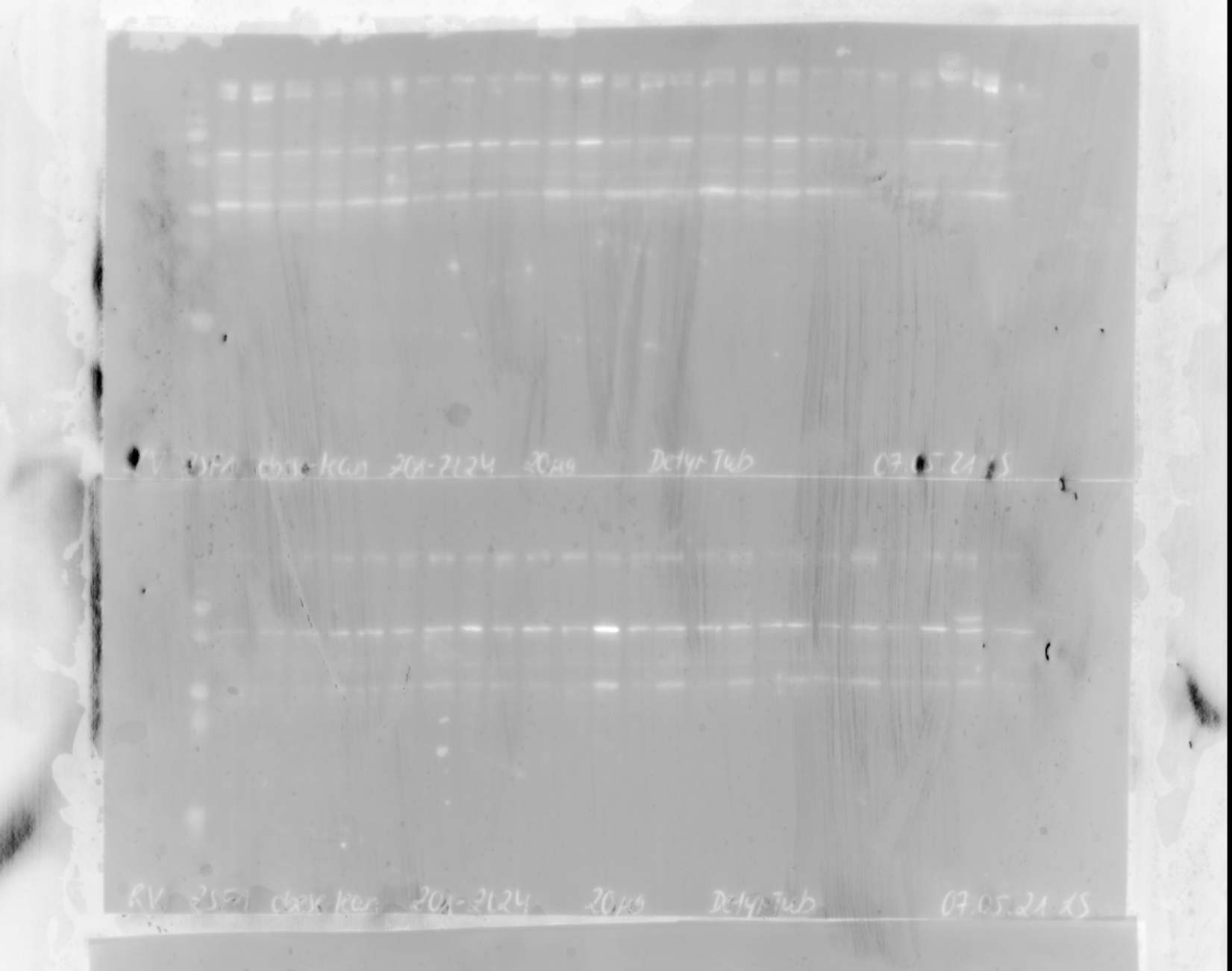


🡨 detyr α-Tub (55 kDa)


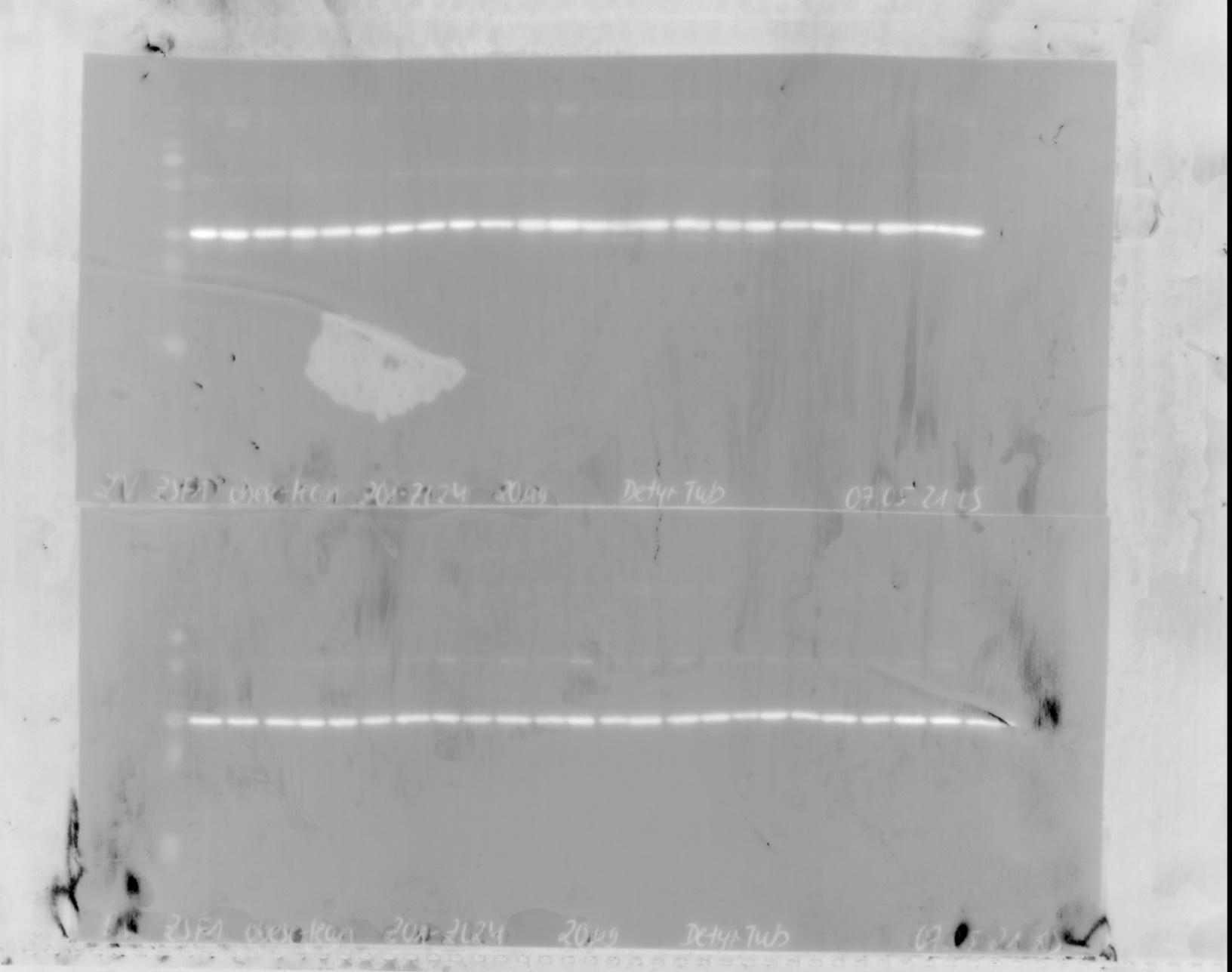


L-ZSF1

O-ZSF1

🡨 GAPDH

(37 kDa)

**D**

L-ZSF1

O-ZSF1


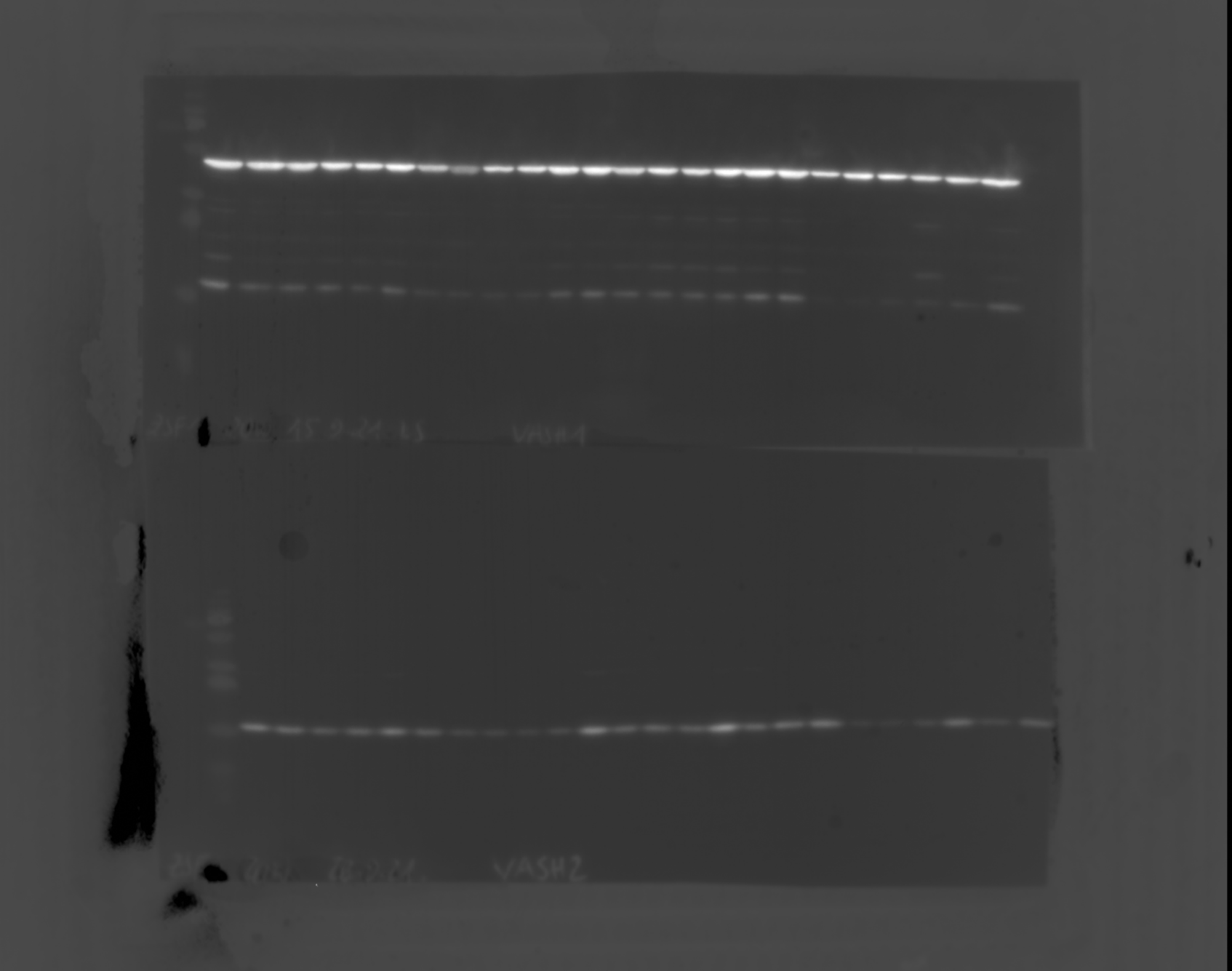


🡨 H2B

(17 kDa)

🡨 VASH1 (41 kDa)

**E**


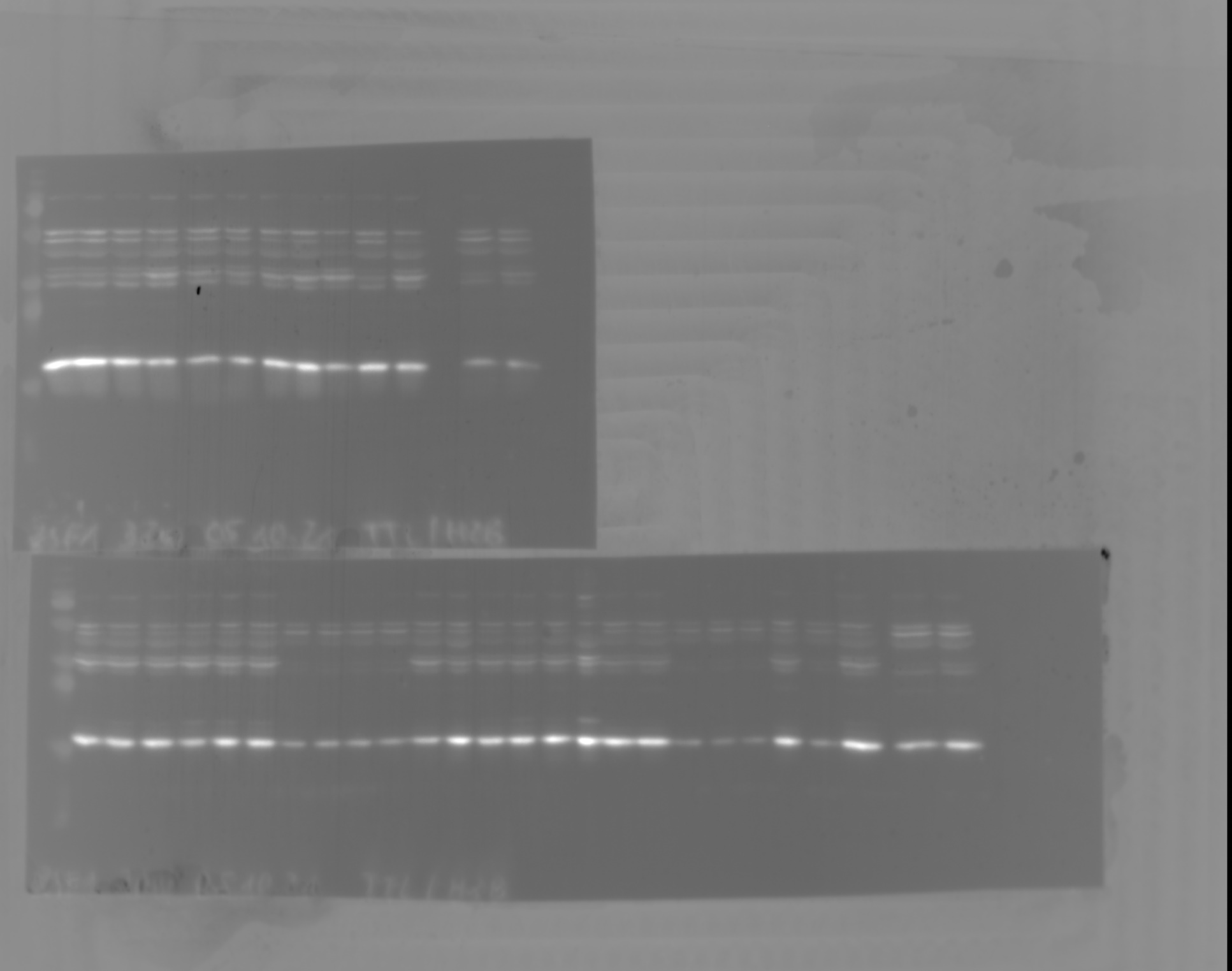


L-ZSF1

O-ZSF1

🡨 H2B (17 kDa)

🡨 TTL (43 kDa)

**Figure 4**: Original Western Blot images of analyses of left ventricles of 32 week old O-ZSF1 (n=6) and L-ZSF1 (n=5) rats (A-E). A - upper panel: α-tubulin, A - lower panel: GAPDH was determined for normalization of α-tubulin, B: tyrosinated α-tubulin (tyr α-Tub) and GAPDH, C: detyrosinated α-tubulin (detyr α-Tub) and GAPDH D: VASH1 and H2B (GAPDH determination was not possible because of comparable protein size with VASH1), E: TTL and H2B. In blots A-C, we also analyzed samples unrelated to the current study. Only the labeled samples are relevant.

**A**


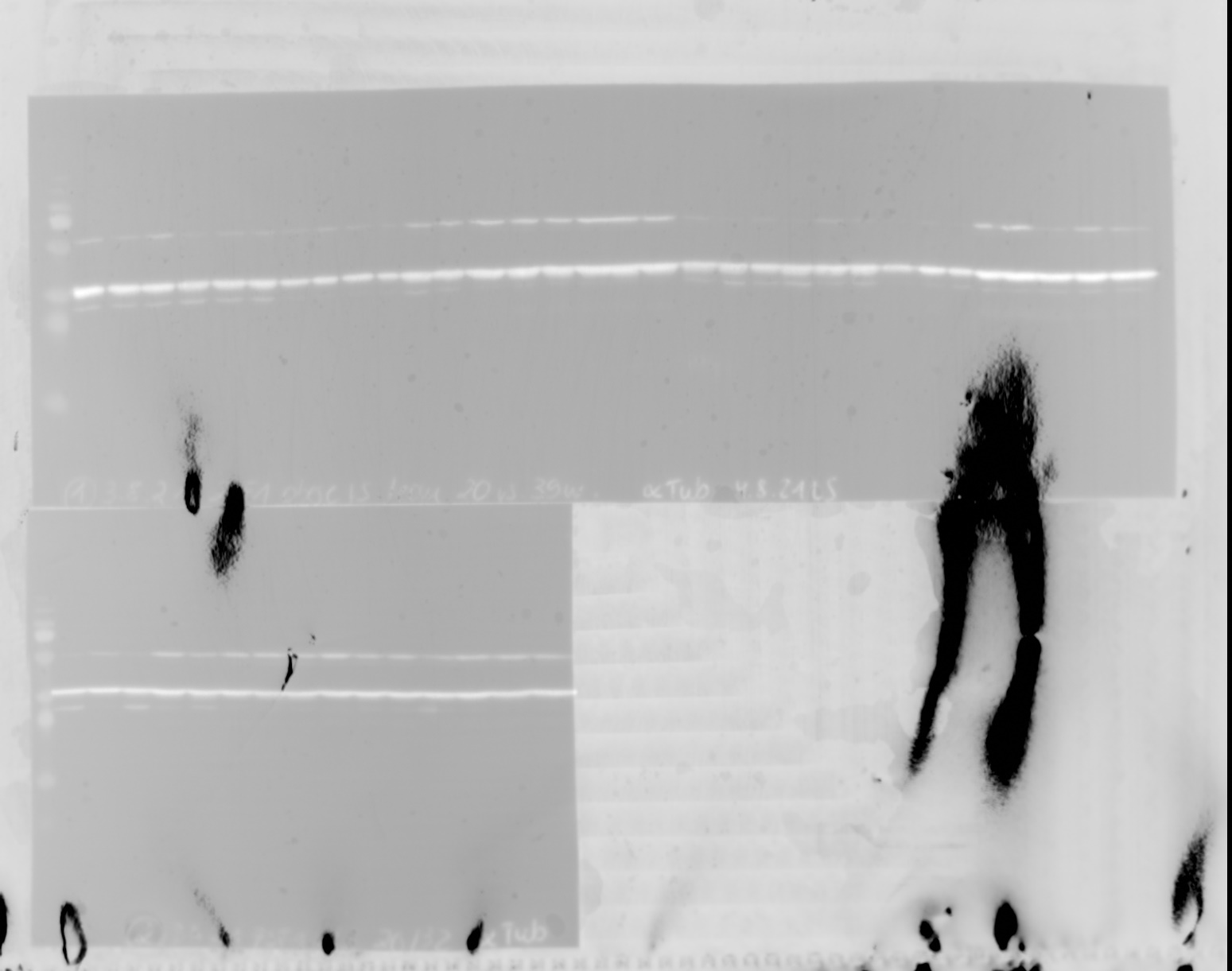


🡨 GAPDH

(37kDa)

🡨 α-Tub (50 kDa)

L-ZSF1

O-ZSF1

**B**


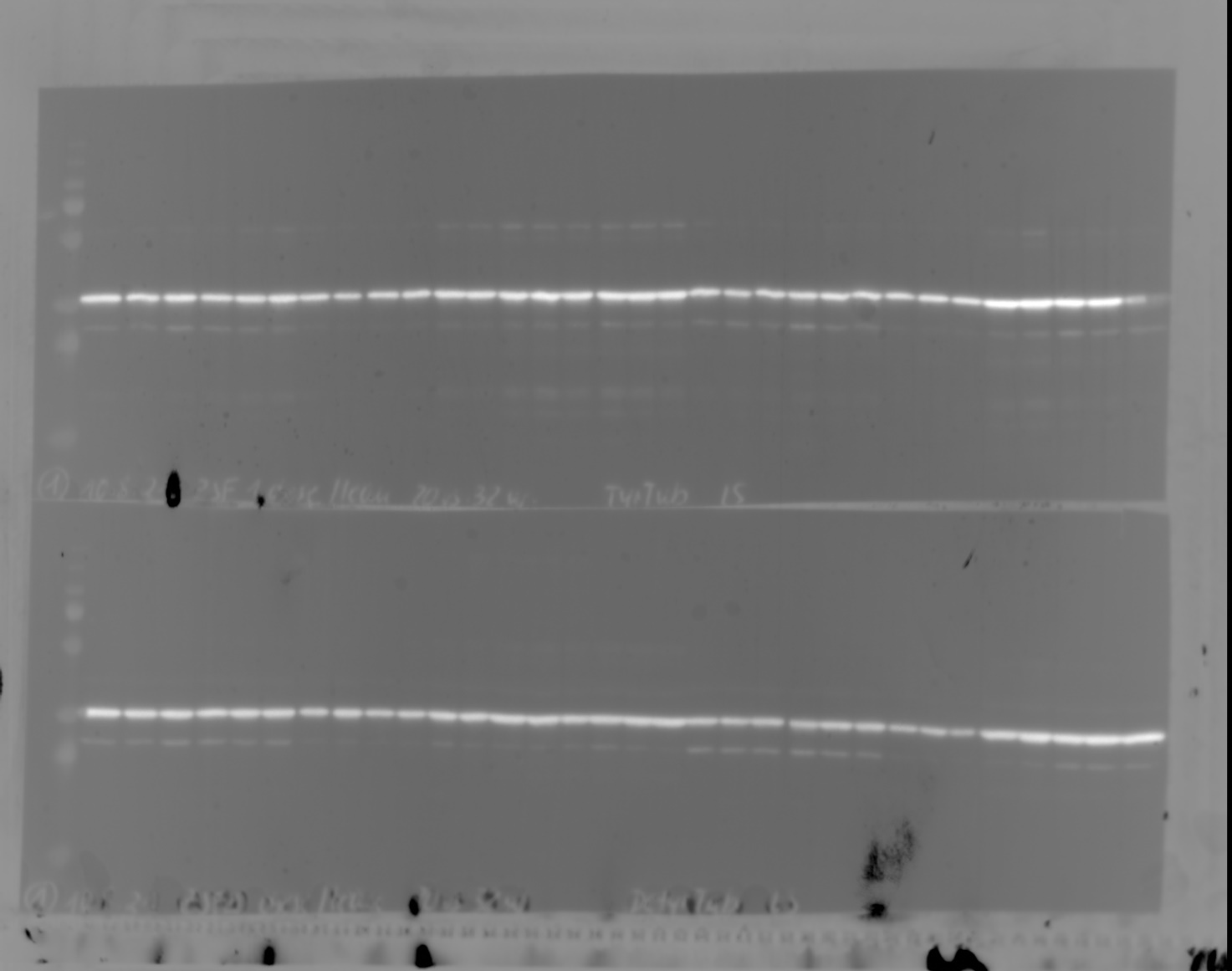


L-ZSF1

O-ZSF1

🡨 tyr α-tubulin

🡨 GAPDH

**C**


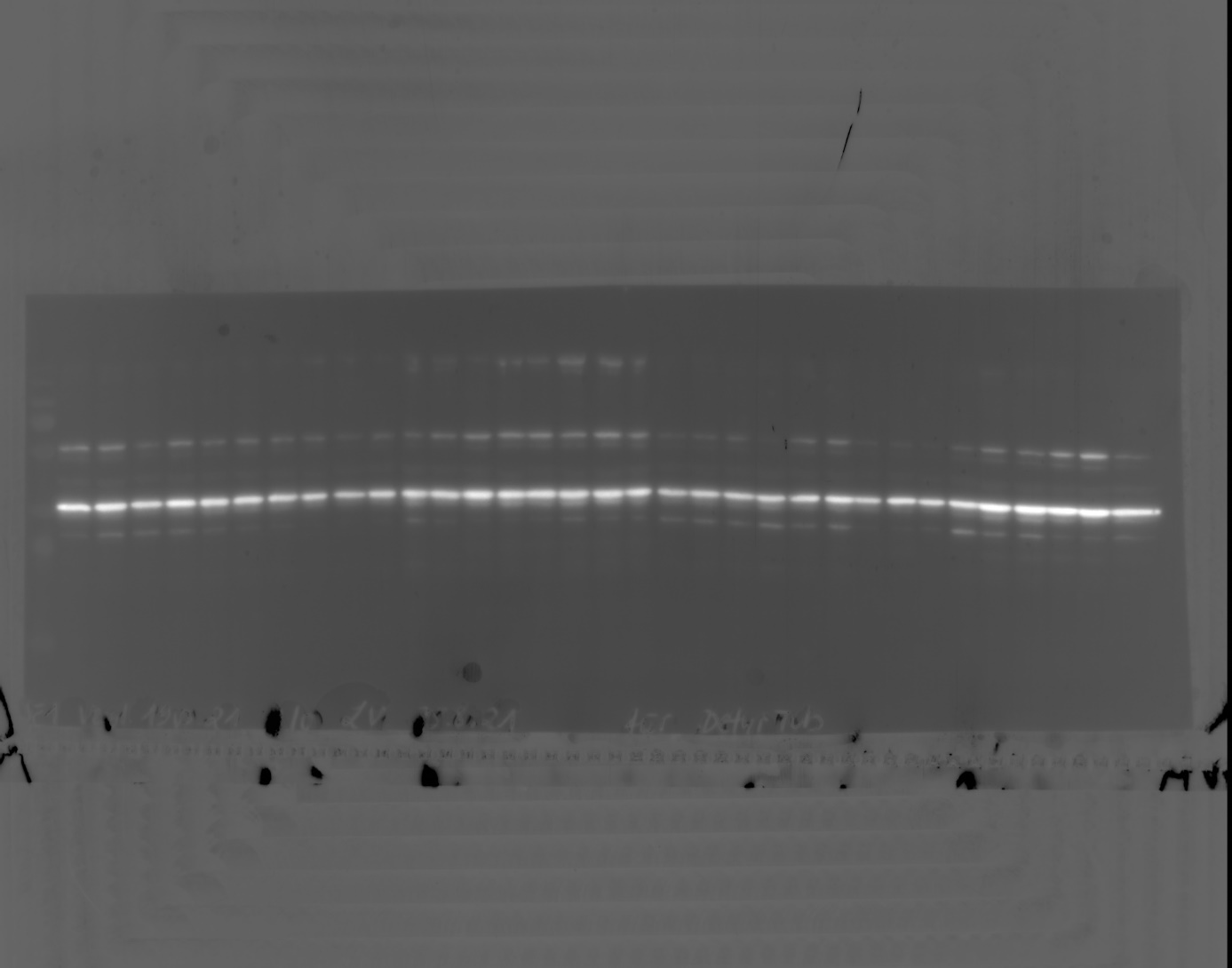


L-ZSF1

O-ZSF1

🡨 detyr α-Tub

🡨 GAPDH

**D**


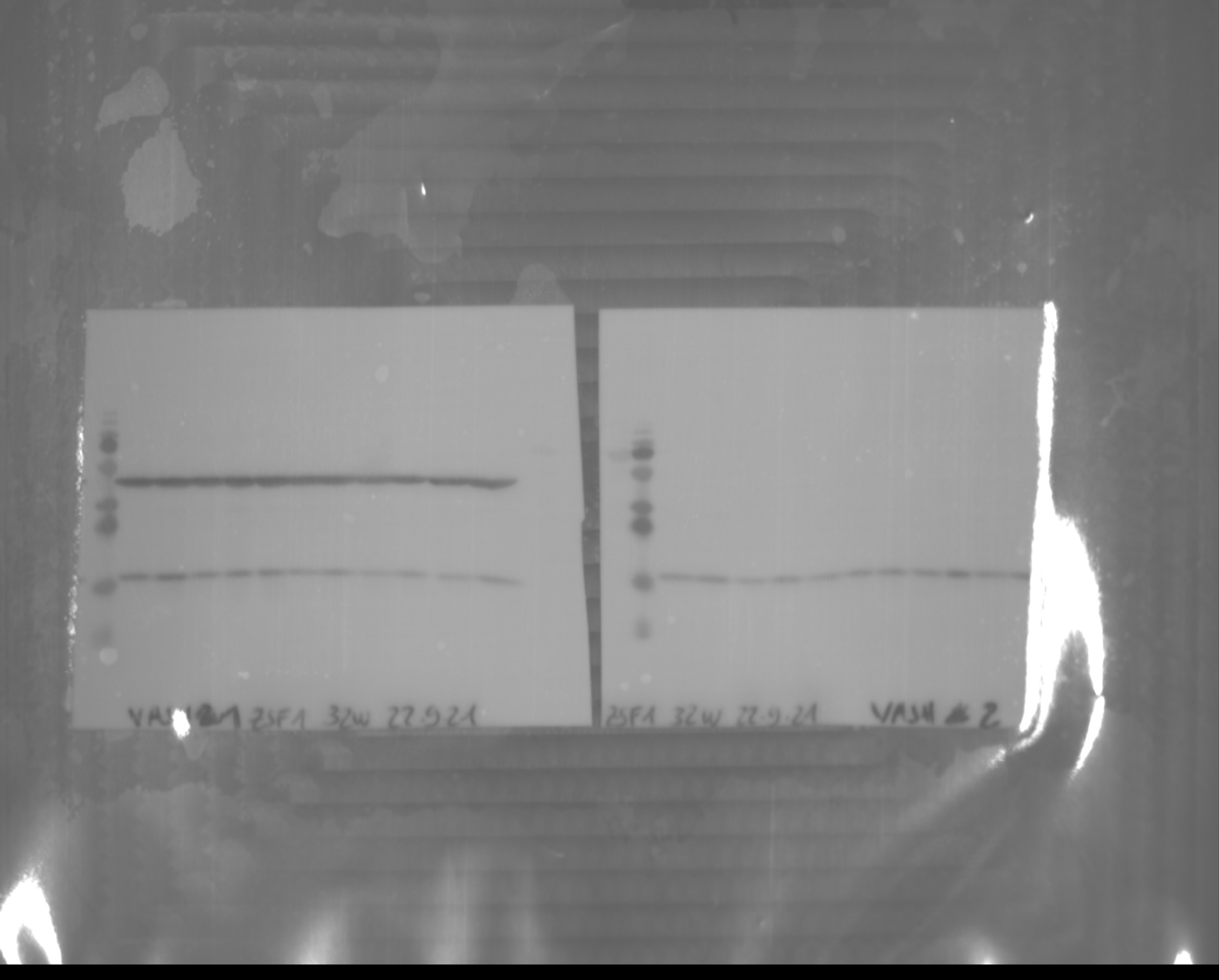


L-ZSF1

O-ZSF1

🡨 H2B (17 kDa)

🡨 VASH1 (41 kDa)

**E**

L-ZSF1

O-ZSF1


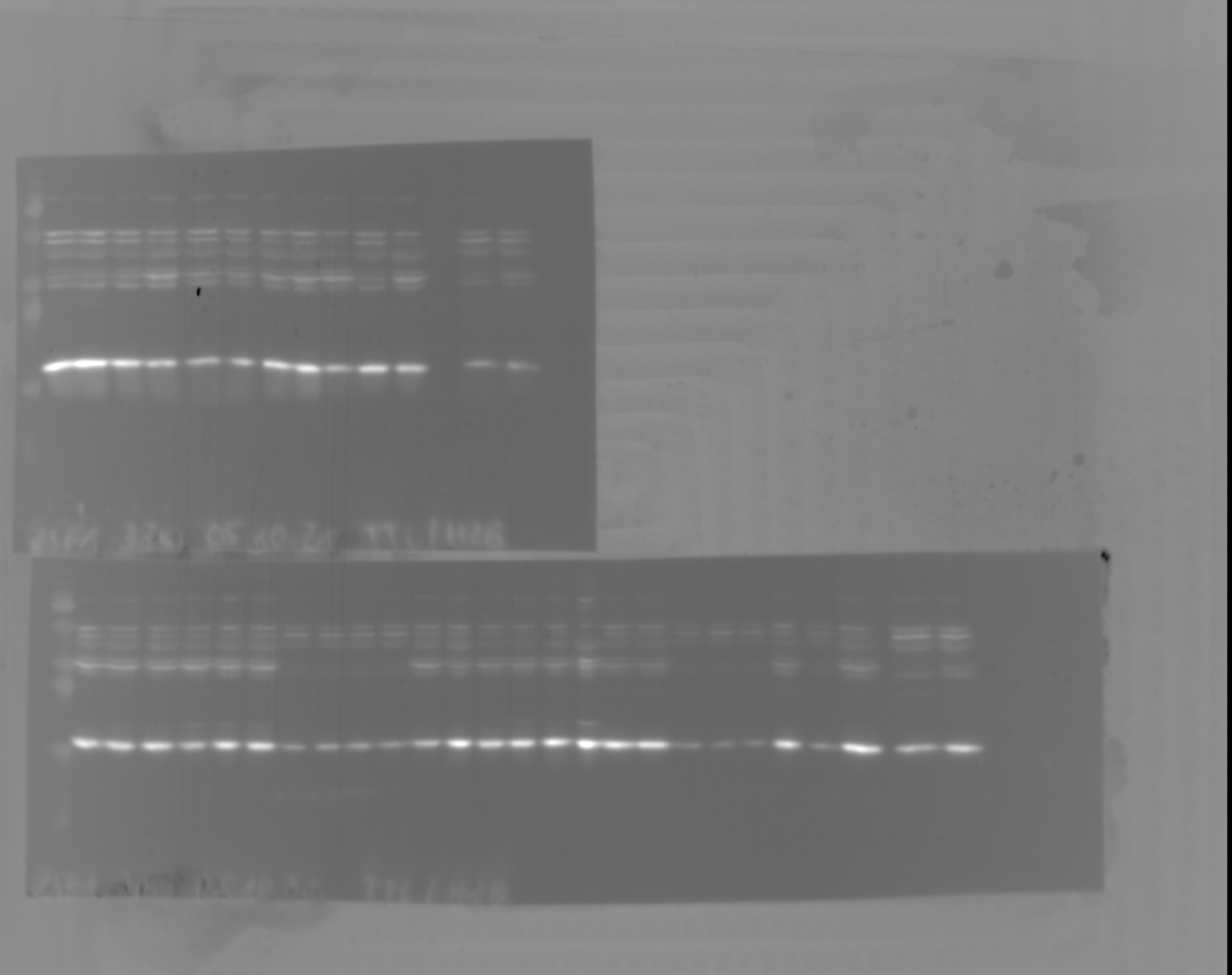


🡨 H2B (17 kDa)

🡨 TTL (43 kDa)
